# Brain distribution of geissoschizine methyl ether in rats using mass spectrometry imaging analysis

**DOI:** 10.1038/s41598-020-63474-x

**Published:** 2020-04-29

**Authors:** Takashi Matsumoto, Yasushi Ikarashi, Mikina Takiyama, Junko Watanabe, Mitsutoshi Setou

**Affiliations:** 1 Tsumura Kampo Research Laboratories, Kampo Research & Development Division, Tsumura & Co., Ibaraki, Japan; 2 Department of Cellular and Molecular Anatomy, Hamamatsu University School of Medicine, Hamamatsu Shizuoka, Japan; 3 International Mass Imaging Center, Hamamatsu University School of Medicine, Hamamatsu Shizuoka, Japan; 4 Department of Systems Molecular Anatomy, Institute for Medical Photonics Research, Preeminent Medical Photonics Education & Research Center, Hamamatsu Shizuoka, Japan

**Keywords:** Biochemistry, Chemical biology

## Abstract

Geissoschizine methyl ether (GM) is one of the main active ingredients responsible for ameliorating the behavioral and psychological symptoms of dementia (BPSD) in Kampo medicine yokukansan. GM is mainly metabolized into hydroxylated forms (HM-1/2). However, the brain distributions of GM and HM has not been reported *in vivo*. In this study, therefore, the plasma concentrations and brain distribution of these compounds were examined *in vivo* using rats injected intravenously with GM. Plasma concentrations were analyzed using liquid chromatography-tandem mass spectrometry analysis and brain distribution using mass spectrometry imaging analysis. Plasma GM and HM-1 concentrations decreased in the 4 h after injection, whereas the concentration of plasma HM-2 increased at 4 h. In the 0.25 h-brain, GM signals were diffusely observed throughout the brain, including the cerebral cortex, hippocampus, striatum, thalamus, amygdala, cerebellum, and cerebral ventricle. HM signals were detected only in the ventricles of the brain at 4 h. These results suggest that plasma GM enters the brain and distributes in the parenchyma of various brain regions involved in BPSD, while plasma HM does not enter the brain parenchyma. This study is also the first to visually demonstrate the brain distribution of GM and its metabolite *in vivo*.

## Introduction

Clinical application of the traditional Japanese Kampo medicine yokukansan (YKS) for treatment of the behavioral and psychological symptoms of dementia (BPSD) has increased in recent years. Research progression the active ingredients and action mechanisms of YKS has increased its clinical applications. Geissoschizine methyl ether (GM) is an alkaloid derived from Uncaria hook, which is a component of YKS. It is considered to be one of the main active ingredients responsible for improving BPSD, such as aggressiveness, irritability, and hallucination^1^. GM has been demonstrated to have multiple actions^2^ and plays roles inserotonergic^3,4^, dopaminergic^3,5^, and adrenergic^6^ neurotransmission as well as in neuroprotection^7,8^.

Previous pharmacokinetic studies have shown that GM can be detected in the plasma and brain of rats^9,10^ and in the plasma of humans^11^ after oral administration of YKS. An *in vitro* cultured blood–brain barrier (BBB) assay showed that GM reached the brain through the BBB^9^. These findings suggest that orally administered GM is absorbed into the blood and enters the brain after passing through the BBB. *In vitro* autoradiographic binding assays in which [^3^H]GM is directly exposed to normal rat brain slices revealed that GM specifically bound to various regions appearing on the surface of brain slices^12,13^. This *in vitro* result suggests that GM binds to the frontal cortex, hippo campus, caudate putamen, amygdala, central medial thalamic nucleus, dorsal raphe nucleus, and cerebellum. Unfortunately, however, GM distribution in the brain after its administration has not been investigated under *in vivo* conditions. To evaluate the distribution of GM in the brain, *in vivo* verification is essential.

Regarding GM metabolites, recent *in vitro* studies using rat and human liver microsomes have demonstrated that GM is metabolized into several metabolites, including hydroxylated, dehydrogenated, hydroxylated + dehydrogenated, demethylated, and hydrated forms^14^. A subsequent *in vitro* study suggested that the hydroxylated forms metabolized by cytochrome P450 3A4 are one of the major metabolites of GM^15^. However, the pharmacokinetics and intracerebral migration of the metabolites remain unstudied.

The purpose of the present study was to directly demonstrate the *in vivo* brain distributions of GM and its hydroxylated metabolite (HM). Mass spectrometry imaging (MSI) using matrix-assisted laser desorption ionization (MALDI)^16–18^ and desorption electrospray ionization (DESI)^19^ represents a powerful tool for visualizing the distributions of biological molecules or metabolites in tissue sections. For example, the liver and kidney distributions of the parent compound and its metabolites in mice orally administered polyphenol (−)-epigallocatechin-3-*O*-gallate have been visualized using MSI analysis^20^. In the present study, therefore, the distributions of GM and its metabolite HM in the brains of rats intravenously injected with GM were investigated by MSI analysis using MALDI and/or DESI. The brain distributions were assessed based on target ingredient concentrations and their time course in the plasma collected from the same animals.

## Results

### GM and its two hydroxylated metabolites, HM-1/2, detected in the plasma

**Figure 1:**
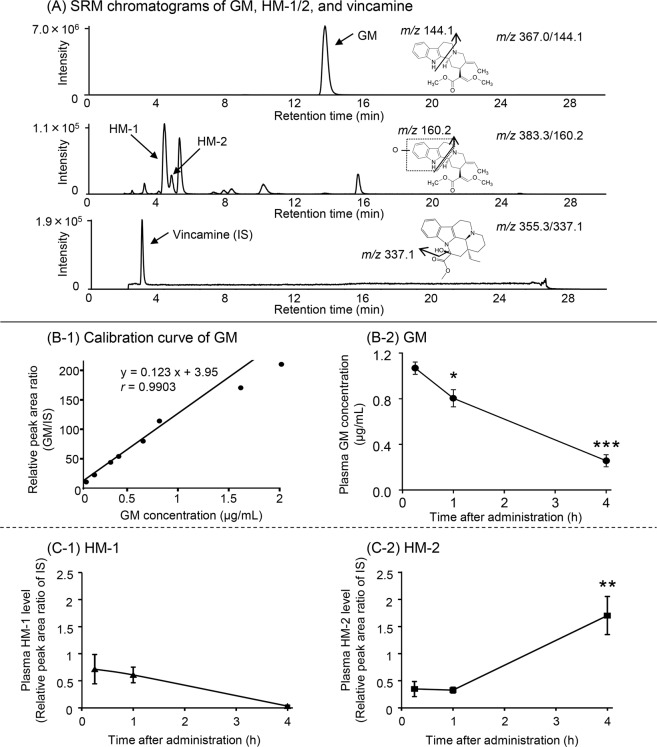
GM and HM-1/2 in the plasma of rats intravenously injected with GM. Blood samples were collected 0.25, 1, and 4 h after intravenous injection of 5 mg/kg GM. (**A**) shows SRM chromatograms of GM (*m/z* 144.1, *t*_R_ 13.6 min) and its two hydroxylated isomers (*m/z* 160.2, *t*_R_: 4.4 min for HM-1 and 4.8 min for HM-2) in plasma collected 0.25 h after injection and SRM chromatograms of vincamine (*m/z* 337.1, *t*_R_ 2.8 min) used as an IS in the analysis of the target compounds. (**B-1**) shows the calibration curve (y = 0.123x +3.95) for GM quantification, indicating high correlation (*r* = 0.9903) between the relative peak area ratio of GM to IS (*y*) and the GM concentration (*x*). HM-1/2 concentrations were expressed as the relative peak area ratio of IS. (**B-2,C-1,C-2**) show the time courses of GM, HM-1, and HM-2 levels in the plasma after GM injection. Each value represents the mean ± standard error of the three animals. *P < 0.05, **P < 0.01, and ***P < 0.001 versus 0.25 h concentration (Dunnett’s test following one-way ANOVA). No statistical significance was observed in the time-dependent changes in plasma HM-1 (one-way ANOVA).

A liquid chromatography-tandem mass spectrometry (LC-MS/MS) analysis was performed to detect GM and HM-1/2 in rat plasma. Figure 1A shows the selected reaction monitoring (SRM) chromatograms of GM, HM-1/2 and vincamine as an internal standard (IS) analyzed from plasma collected 0.25 h after intravenous GM injection. Specific fragment ions of GM (*m/z* 144.1, *t*_R_ 13.6 min) and two metabolite isomers with the same fragment ion (*m/z* 160.2) at different *t*_R_ (4.4 min for HM-1 and 4.8 min for HM-2), as well as vincamine (*m/z* 337.1, *t*_R_ 2.8 min), were detected in the plasma.

**Figure 2:**
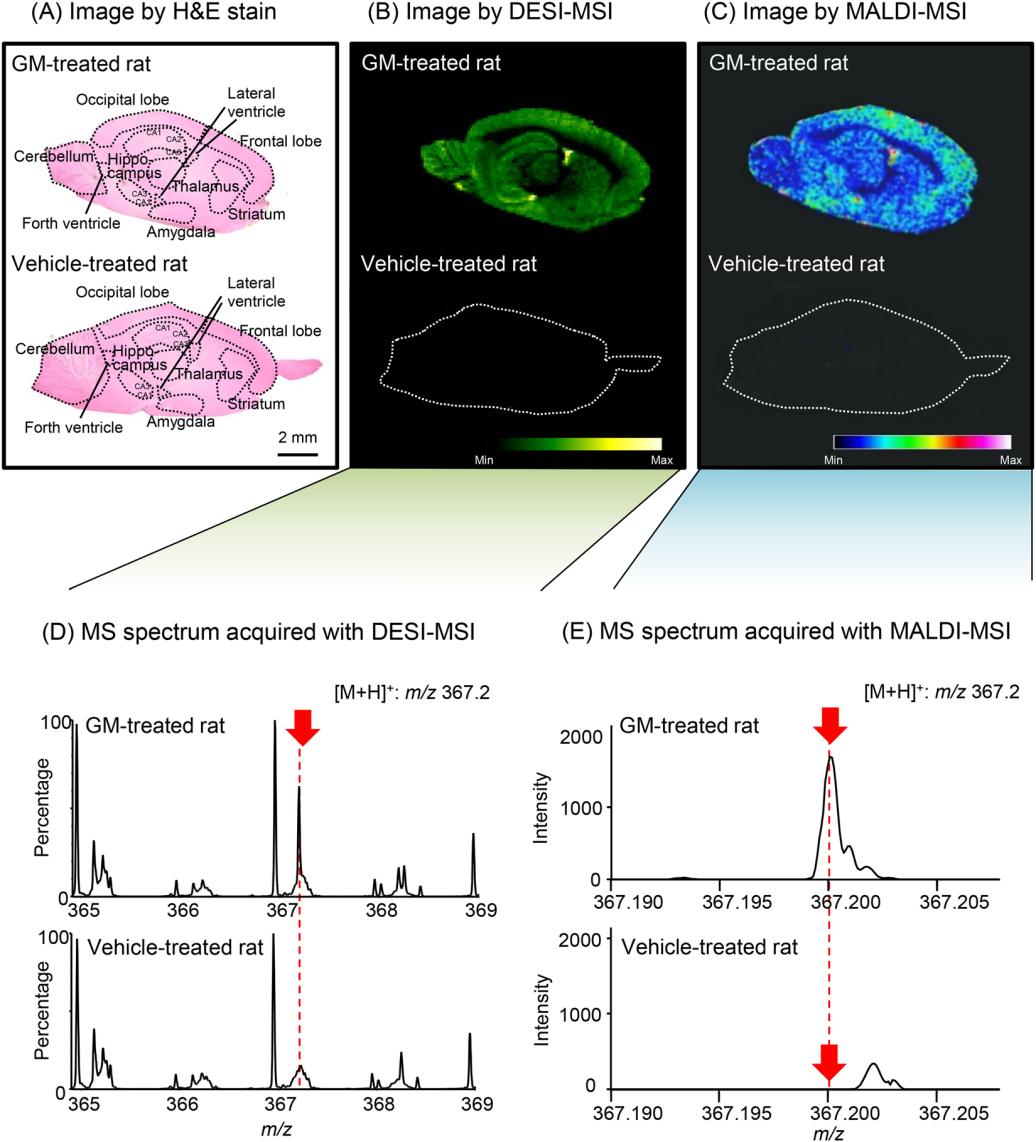
DESI-MSI or MALDI-MSI analysis of GM distributed in rat brains. The upper and lower panels in (**A–C**) show typical images of the sagittal sections obtained with H&E staining and DESI-MSI or MALDI-MSI analysis in the brains of rats sacrificed 0.25 h after intravenous injection with GM (5 mg/kg) or vehicle (0.6 mL/kg of 70% DMSO as control). The scale bars show 2 mm. Ion images of the protonated GM signal ([M + H]^+^* m/z* 367.2) were acquired with a spatial resolution of 200 μm for both DESI-MSI and MALDI-MSI. In the DESI-MSI analysis, the variance of intensity among scan points was corrected by normalization to the total ion count at each scan point. (**D,E**) show MS spectra acquired with DESI-MSI and MALDI-MSI analyses in the same brain sections shown in (**B,C**).

Figure 1B-1 shows a calibration curve for quantifying GM, and Fig. 1B-2 shows a time course of plasma GM concentration calculated using the calibration curve. The GM concentration, which was 1.07 µg/mL at 0.25 h postinjection, rapidly decreased in a time-dependent manner over 4 h [one-way analysis of variance (ANOVA): *F*(2, 6) = 45.5, *P* < 0.001]. Dunnett’s post-hoc test showed that the plasma concentrations at 1 and 4 h were significantly lower than the 0.25 h value. The pharmacokinetic parameters of GM calculated by one-compartment analysis were as follows: an elimination rate constant of 0.4 h^−1^, an apparent elimination half-life of 1.8 h, a total clearance of 1.7 L/h/kg, and a distribution volume of 4.4 L/kg.

Figure 1C-1,C-2 show the time courses of HM-1 and HM-2 levels, respectively, in the plasma after GM injection. The HM-1/2 levels were represented as the relative peak area ratio of the IS, as there are currently no standard products for measuring these compounds. The HM-1 levels decreased in a time-dependent manner, but were not significantly different [one-way ANOVA: *F*(2, 6) = 4.27]. Conversely, metabolite isomer HM-2 levels showed significant differences in time factor [one-way ANOVA: *F*(2, 6) = 12.8, *P* < 0.01]. Dunnett’s post-hoc test showed that there was no significant difference between the 0.25 h and 1 h levels, but that the 4 h level was significantly higher (*P* < 0.01) than the 0.25 h level.

### Comparison between MALDI and DESI-MSI images of GM in the brain

Figure 2 shows typical images by hematoxylin and eosin (H&E) staining (A), DESI-MSI (B), and MALDI-MSI (C) and MS spectra acquired with DESI-MSI (D) and MALDI-MSI (E) analyses in sagittal brain sections. The upper or lower panel in the images and the spectra show the analytical data of rat brain sections collected 0.25 h after intravenous injection of GM (5 mg/kg) or 70% dimethyl sulfoxide (DMSO) (vehicle: 0.6 mL/kg), respectively. The cerebral cortex (including the occipital and frontal lobes), hippocampus, striatum, thalamus, amygdala, cerebellum, and cerebral ventricle (including the lateral and forth ventricles) were identified in the H&E-stained sections. In both the DESI-MSI and MALDI-MSI images, a diffuse GM ion signal was observed throughout the brain parenchyma of GM-treated rats. In particular, the signal intensity in the lateral ventricle was higher than that in other parenchymal areas. On the other hand, no signal was observed in the brain sections of vehicle-treated control rats.

### Distributions and time courses of GM and HM in the brain

#### GM

**Figure 3:**
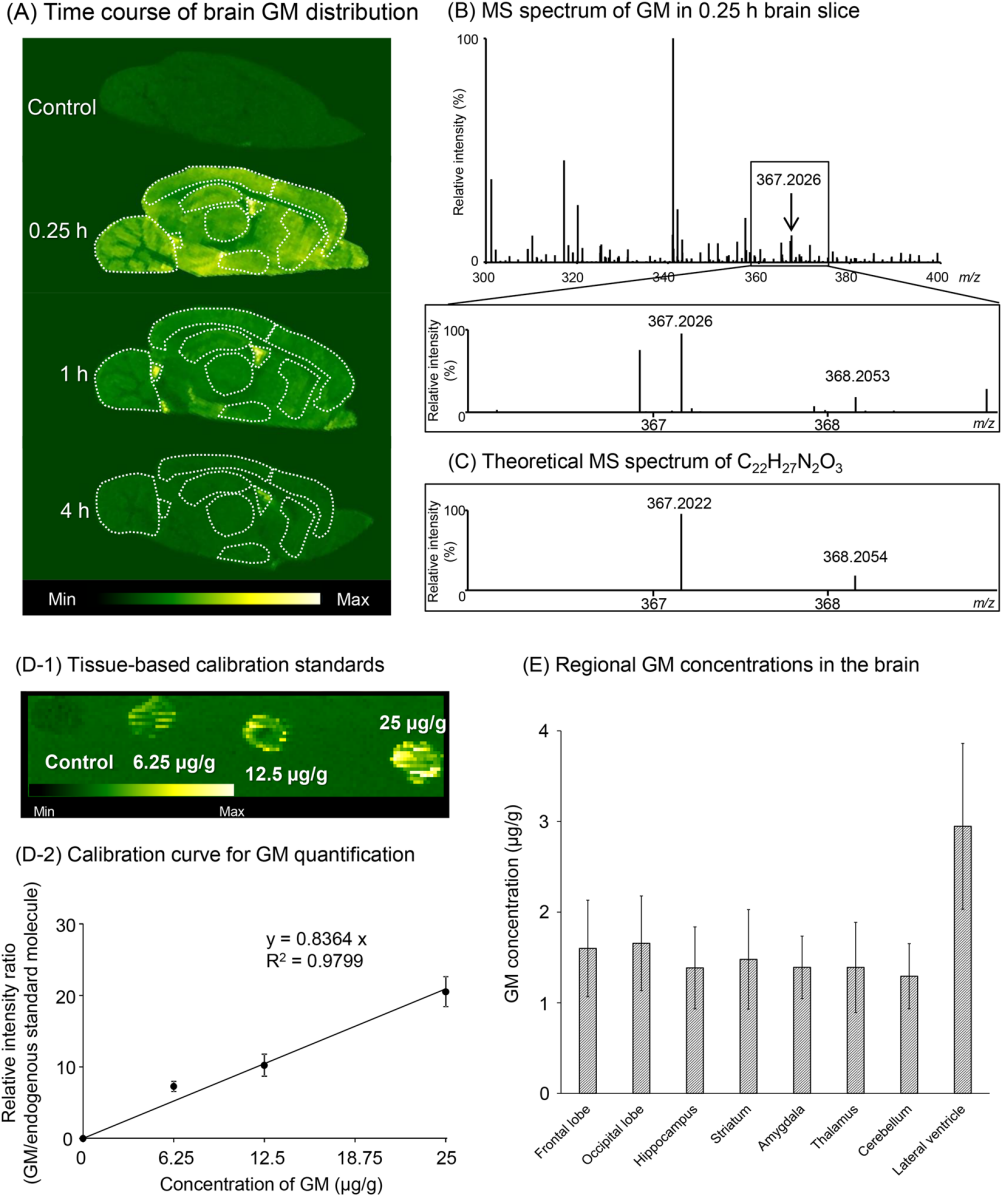
GM distribution in rat brains using DESI-MSI analysis. (**A**) shows the time course of GM distribution in the brains of rats intravenously injected with GM. GM-treated rats were sacrificed 0.25, 1, and 4 h after GM injection (5 mg/kg). Control rats were sacrificed after intravenous injection of vehicle (70% DMSO: 0.6 mL/kg). (**B**) shows the MS spectrum of GM detected in the lateral ventricle of a 0.25 h brain slice. (**C**) shows the theoretical MS spectrum of a compound (C_22_H_27_N_2_O_3_) with the same chemical composition as protonated GM obtained automatically by elemental composition analysis. (**D-1**) shows the typical tissue-based calibration standards. Frozen sections of brain homogenates spiked with known quantities of GM (6.25–25 μg/g) were used to prepare a calibration curve for GM quantification. (**D-2**) shows the calibration curve for GM quantification. The signal intensity ratio of GM to an endogenous standard molecule (*m/z* 366.95) showed a linear regression curve with a good correlation coefficient (*R*^2^ = 0.9799). Each value represents the mean ± standard error of the four sections. (**E**) shows regional GM concentrations in 0.25 h brain images. Each value represents the mean ± standard error of the three animals. No statistical significance was observed among regions (one-way ANOVA). All values used in the calculations of GM concentrations are summarized in Supplementary Table S1.

Figure 3A shows the time course of the GM distribution visualized by DESI-MSI analysis in the brains of rats intravenously injected with GM (5 mg/kg). Compared with the control brain image, distinct diffuse GM intensities were reconfirmed in a 0.25 h brain section, as shown in Fig. 2B. Intracerebroventricular GM intensity was higher than the intensity in other parenchymal areas and was also detected 1 and 4 h after the GM intensity in the parenchyma had almost disappeared. To illustrate the reproducibility of the DESI-MSI analysis, GM distribution images analyzed repeatedly between 0.25 and 4 h are shown in Supplementary Fig. S1.

Figure 3B shows the MS spectrum of GM detected in the lateral ventricle of a 0.25 h brain slice. Protonated ions ([M + H]^+^, *m/z* 367.2026) of GM and its isotope ions ([M + H + 1]^+^, *m/z* 368.2053) were detected. The intensity ratio (GM/isotope) and their exact mass almost exactly agreed with the theoretical values of a compound (C_22_H_27_N_2_O_3_) with the same chemical composition as protonated GM, which was automatically obtained by elemental composition analysis (Fig. 3C). In other words, the ratio of the ppm error of the detected peak to the theoretical peak of protonated GM was 1.1. In addition, the MS/MS analysis showed dissociation of a specific fragment ion (*m/z* 144.079) from the protonated GM ion (Supplementary Fig. S2).

Figure 3D-1 shows the tissue-based calibration standards: GM signal intensities on frozen sections of brain homogenates spiked with known quantities of GM (6.25–25 μg/g) increased in a concentration-dependent manner. When the GM signal intensity was expressed as a ratio relative the intensity of an endogenous standard molecule (*m/z* 366.95) that is stably and uniformly detected in the brain (Supplementary Figs. S3 and S4), a high correlation (correlation coefficient *R*^2^ = 0.9799) was found between the GM concentration and the intensity ratio (Fig. 3D-2). Using this calibration curve, the regional concentrations of GM in control and 0.25-h brain images were quantified (Fig. 3E). GM was not detected in any region of the brains of control rats. However, in brain sections obtained 0.25 h after injection, GM was detected in the cerebral cortex (the frontal and occipital lobes), hippocampus, striatum, amygdala, thalamus, cerebellum, and lateral ventricle in the concentration range of 1.3–2.9 μg/g. The lateral ventricle was clearly observed in all slice specimens, but unfortunately some specimens failed to show the fourth ventricle in the same section, probably due to a technical error in the sectioning process of the slice specimen. However, in sections where both ventricles were clearly visible, similarly high signal strength was observed for both. Therefore, for quantification of ventricular signals, we selected the lateral ventricle that was reliably observed in all sections as a representative of the cerebral ventricles. The lateral ventricular GM concentration was higher than in the other regions, but no significant difference was observed between the other regions [one-way ANOVA: *F*(7, 16) = 0.98].

#### HM

**Figure 4:**
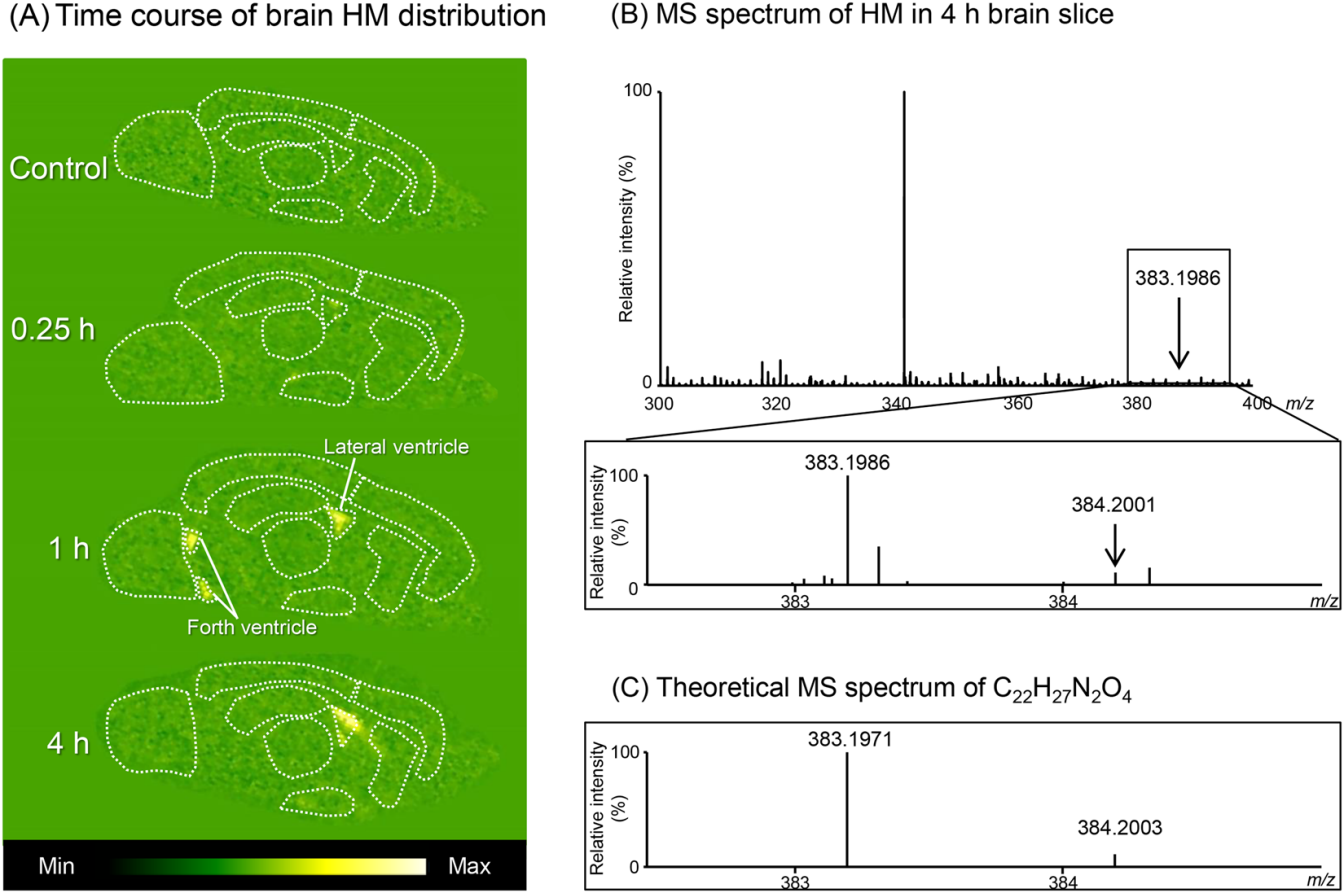
HM distribution in rat brains using DESI-MSI analysis. (**A**) shows the time course of brain HM distribution in rats intravenously injected with GM. HM analysis was performed on the same brain sections used for GM analysis. (**B**) shows the MS spectrum of HM detected in the lateral ventricle of a 4 h brain slice. (**C**) shows the theoretical MS spectrum of a compound (C_22_H_27_N_2_O_4_) with the same chemical composition as protonated HM obtained automatically by elemental composition analysis.

Figure 4A shows the time course of HM distribution visualized by DESI-MSI analysis in the brains of rats intravenously injected with GM (5 mg/kg). The HM images correspond to the total intensity of both HM-1 and HM-2 protonate ions (*m/z* 383.2) because it was impossible to separate HM isomers in this analysis, unlike LC-MS/MS analysis that employs LC separation. Similar to the control brain image, the HM signal was below the detection limit in any region on the 0.25 h brain section. However, pronounced signals were detected only in the cerebral ventricle, including the lateral and forth ventricles, on the 1 h brain image, after which the intensity in the lateral ventricle of the 4 h brain section was stronger than that at 1 h. In the brain parenchyma, no HM signal was detected even in the 4 h brain section. To demonstrate the reproducibility of the DESI-MSI analysis, HM distribution images analyzed repeatedly between 0.25 and 4 h are shown in Supplementary Fig. S1.

Figure 4B shows the MS spectrum of HM detected in the lateral ventricle of the 4 h brain slice. The specific protonated ion ([M + H]^+^, *m/z* 383.1986) of HM and its isotope ion ([M + H + 1]^+^, *m/z* 384.2001) were detected. The intensity ratio (HM/isotope) and the exact mass almost exactly agreed with the theoretical values of protonated HM (C_22_H_27_N_2_O_4_) obtained by elemental composition analysis (Fig. 4C). In other words, the ratio of the ppm error of the detected peak to the theoretical peak of protonated HM was 3.9.

## Discussion

YKS has been reported to ameliorate neuropsychiatric symptoms such as anxiety and aggressive behaviors by oral administration of a 1 g/kg dose in various animal models^4,21–24^. Since GM is detected in the plasma and brains of rats orally administered this YKS dose^9,10^, it is believed that GM contained in YKS is absorbed into the blood and reaches the brain. However, to reliably visualize the brain distribution of GM by MSI analysis, it is necessary to increase its plasma concentration. Therefore, in the present study, a high dose (5 mg/kg) of GM, which corresponds to approximately 30 to 40 times the GM content in 1 g of YKS^4^, was directly injected intravenously. Upon injecting this dose, the plasma concentration after 0.25 h rose up to approximately 500 times the maximum concentration value 1 h after oral administration of 1 g/kg YKS^9^, and thereafter, rapidly decreased through 4 h (Fig. 1). For the first time, this study showed changes in plasma concentrations after intravenous injection of GM. The pharmacokinetic parameters calculated in our one-compartmental analysis can help to understand the pharmacokinetic characteristics of GM. Among these, GM was found to have a high total clearance value (1.7 L/h/kg), suggesting that GM can easily be converted into metabolites, such as hydroxylated forms, or is easily excreted into bile and/or urine. The apparent volume of distribution (4.4 L/kg) calculated in the present study with intravenous injection of GM dissolved in DMSO was similar to that obtained in previous study using rats orally administered YKS without DMSO^25^. This result suggests that DMSO dose not affect BBB permeability of GM.

In addition, in a previous study of oral administration of YKS containing GM to rats (DMSO was not used in this study), GM was detected in the brain and its concentration varied in parallel with plasma concentration. In both cases, the *t*_max_ was 30 minutes after YKS administration^10,25^. An *in vitro* BBB permeability study using cultured cells showed the permeability of GM is comparatively high^9^. These results suggest that GM in plasma easily passes through the BBB. The present study, in which GM dissolved in DMSO was directly injected intravenously, showed that brain GM concentrations varied in parallel with plasma concentrations. This result was similar to the YKS study given orally without DMSO described above, suggesting that BBB permeability of GM was not due to the addition of DMSO. Neuwelt *et al*. (1983) and Ziylan *et al*. (1988) also previously demonstrated that rats injected intravenously with high concentrations of DMSO did not show significant barrier opening^26,27^. This result also strongly supports our idea.

We first examined whether GM could be detected by MALDI or DESI-MSI analysis in the brain collected 0.25 h after injection, at which time the plasma GM concentration was the highest, as described above. This comparative study showed that GM signals could be detected, and the distributions of GM in the images were almost similar for both methods (Fig. 2). Additionally, the mass errors in ppm between the detected and theoretical peaks of GM were low in both the MALDI-MSI (5.4 ppm) and DESI-MSI (1.1 ppm) analyses. However, for the MALDI-MSI method, target molecules must be ionized under a vacuum after a tissue slice is coated with a matrix to ionize the molecule^28^. The DESI-MSI method developed as an alternative does not require the matrix and can ionize molecules directly under atmospheric pressure^29^. Therefore, the DESI-MSI method is considered to simplify the workflow^19^. Because of the convenience of the DESI-MSI method, we selected the DESI-MSI method as a tool for visualization of the distributions of GM and its metabolite HM in brain sections.

To verify that the DESI-MSI signal of GM detected in the brain is specific, MS/MS analysis of the signal was performed. As a result, the specific fragment of the GM ion was detected (Supplementary Fig. S2). In addition, elemental composition analysis supported that the signal detected by DESI-MSI corresponded to the same chemical composition of GM. These results suggest that the GM signal detected by DESI-MSI is indeed GM. To objectively evaluate the brain distribution of GM signals visualized on DESI-MSI images, we sought to quantify the regional GM concentration on the brain images. The calibration curve for quantifying regional GM concentration was prepared using brain homogenates with various concentrations of GM standard. Calibration curves developed using such tissue-based calibration have been demonstrated to be suitable for the quantification of small molecule drugs in biological tissue sections by MSI^30^. Furthermore, in the present study, the interscan variability of the GM signal intensity was adjusted by measuring the relative intensity to an endogenous standard molecule (*m/z* 366.95) detected stably and uniformly in brain tissue (Supplementary Figs. S3 and S4). Unfortunately, this molecule could not be identified, but the uniform distribution of the *m/z* 366.95 molecule in the brain suggests that this molecule is useful as an endogenous internal standard for MS imaging analysis. In the future, it will be necessary to identify this molecule and understand the physiological role.

As a result of using the endogenous standard molecule, a high correlation (*R*^2^ = 0.9799) was found between GM concentrations and intensity ratios (Fig. 3D-2). Both the quantification data using the tissue-based calibration curve and the DESI-MSI image of GM demonstrated that GM was diffusely distributed throughout the brain parenchyma, including the cerebral cortex, hippocampus, striatum, thalamus, amygdala, and cerebellum. In contrast, the intensities of ventricular GM (Figs. 2B, 3A,E) and HM (Fig. 4A) were higher than in the brain parenchyma. To confirm whether the higher intensity of GM and HM in the ventricle was due to ion suppression by the coexisting matrix component, vincamine (which has a similar structure to GM) was examined in the brain sections using DESI-MSI analysis. Results showed that vincamine signals were uniformaly detected in the brain parenchyma but was considerably lower in the ventricles (Supplementary Fig. S4). This result suggests that the signal intensities of GM and HM in the ventricle were not due to ion suppression but to their high concentrations.

This is the first report to visually show the *in vivo* results of GM distribution in the brain. A previous *in vitro* autoradiographic binding assay demonstrated that [^3^H]GM bound to dopamine D_2_, adrenergic α_2A_, and μ-opioid receptors; L-type Ca^2+^channels; and 5-HT_1A_, 5-HT_2A_, 5-HT_2B_, 5-HT_2C_, and 5-HT_7_ receptors in various regions of the brain slices^1,12^. Based on the combined results of DESI-MSI and autoradiographic binding analyses, GM is believed to be distributed uniformly in the brain, and it exerts pharmacological effects by binding to various channels and receptors.

The time-dependent change in GM concentration in each brain region was similar to that in the plasma: the highest concentration in the brain was observed at 0.25 h (i.e., the time of the highest plasma concentration) after injection and subsequently decreased in parallel with the plasma concentration. In addition, we found that the intracerebroventricular GM concentration was higher than that in the brain parenchyma at 0.25 h after injection, and remain higher even after 1 and 4 h, by which time the concentration in the parenchyma had almost disappeared. There are two conceivable routes that drugs in the blood, including GM, can take to reach the brain parenchyma. One route is the transfer of the drug from brain capillaries to the parenchymal interstitial fluid (ISF) through the blood-brain barrier (BBB)^31^. The other route is the passing of drugs from the choroid plexus artery to the cerebrospinal fluid (CSF) through the blood-CSF barrier (BCSFB) and, then, through the CSF-brain barrier (CSFBB) to the parenchymal ISF^32^. As the surface area of the BBB is 5000 times larger than that of the BCFSB, the BBB is considered to be a superior drug transport route^33^. The distribution volume value of 4.4 L/kg, which was one of the GM pharmacokinetic parameters obtained in the present study, suggests that GM has comparatively high transitivity to organs. In addition, GM was already detected in the brains of rats after oral YKS administration^9,10^. An *in vitro* BBB permeability study using cultured cells demonstrated that GM is able to cross the BBB^9^. Furthermore, drugs that have passed through the BBB have been considered to be able to easily reach brain neurons because brain capillaries constituting the BBB circulate throughout the brain in a mesh-like form^33^. Taken together, these findings suggest that plasma GM enters the brain via the BBB and is diffusely distributed in various brain regions. One possible reason for the diffuse distribution of GM over various brain regions may be the capillary distribution in the brain. In addition, the result that a high concentration of GM was observed in the ventricle early after injection suggests that GM may enter the CSF directly, not only via the BBB, but also via the BCSFB, although a detailed examination is necessary in the future.

On the other hand, the hydroxylated form, which is the main metabolite of GM, was detected in the plasma as two isomers, namely, HM-1 and HM-2, as we estimated in previous *in vitro* drug metabolism studies^14,15^. Generally, drugs injected intravenously - unlike oral administration - are not considered to have undergone intestinal metabolism. In other words, the GM metabolite HM detected in the plasma after intravenous GM injection can be considered to have been mainly converted by hepatic metabolism. However, the time-dependent changes in the plasma concentrations of each isomer exhibited the opposite behavior. Specifically, HM-1 levels decreased and HM-2 levels increased in a time-dependent manner (Fig. 1C). In this study, plasma HM-1 and HM-2 levels were expressed as signal intensities, as there are currently no standards to quantify HM-1 and HM-2 concentrations. The signal intensity of these hydroxylated metabolites was much lower than that of GM. This comparison suggests that HM concentrations may be much lower than GM concentrations. In any case, the reason for the difference in the pharmacokinetics of the two metabolites remains unclear. Several factors, such as liver metabolism and renal clearance, are presumed to be involved. Further studies are needed to elucidate the pharmacokinetic differences between HM-1 and HM-2 and their pharmacological effects.

In the brain slices, the HM isomers were detected and visualized as a hydroxylated form of HM because it is impossible to distinguish between these isomers by DESI-MSI analysis. The signal was observed only in the cerebral ventricle from 1 h after injection, and the intensity after 4 h was stronger than that at 1 h. Unfortunately, a specific fragment of HM could not be verified in the MS/MS analysis due to the low absolute intensity of the precursor ion. However, elemental composition analysis indicated that the signal had the same chemical composition as HM, suggesting that the signal detected by DESI-MSI is probably HM. The time-dependent change in HM intensity observed in the ventricle was similar to that of HM-2 in the plasma, suggesting that the hydroxylated form in the ventricle might reflect HM-2. In addition, unlike GM, the result that HM was detected in the ventricle without being detected in the brain parenchyma suggests that plasma HM may have entered the CSF directly via the BCSFB, not through the BBB; however, the detailed mechanism must be examined in the future. In DESI-MSI analyses of the brain slices of GM-treated rats, we attempted to detect various GM metabolites identified in previous *in vitro* GM metabolism studies^14,15^; however, we were unable to detect metabolites other than HM.

The CSF produced in the choroid plexus circulates in the cerebral ventricles and is absorbed into the venous blood via arachnoid granulation and protrudes into the dural venous sinuses^34^. Therefore, we assume that GM and HM in the CSF are also eventually excreted into the venous blood through this system and metabolized, although the excretion mechanism is still unknown.

In conclusion, the present study is the first to visually demonstrate the distributions of GM and HM in the brains of rats intravenously administered GM via DESI-MSI analysis. The results strongly suggest that plasma GM absorbed after the oral administration of YKS enters the brain through the BBB and distributes into various brain regions involved in the behavioral and psychological symptoms of dementia.

## Materials and Methods

### Drugs and reagents

GM (Lot No. GME001) was supplied by the Botanical Raw Materials Research Department of Tsumura & Co (Ibaraki, Japan). DMSO (a solvent for the test substance) was purchased from Wako Pure Chemical Industries, Ltd. (Osaka, Japan), vincamine (IS for measuring GM and HM-1/2) from Tokyo Chemical Industry Co., Ltd. (Tokyo, Japan), and 2,5-dihydroxybenzoic acid (a matrix for MALDI-MSI analysis) from Bruker Daltonics (Bellerica, MA, USA). Other chemicals were purchased from commercial sources.

### Animals

Seven-week-old female Wistar rats were purchased from Charles River Laboratories (Yokohama, Japan). The animals were housed under a temperature of 23 ± 3 °C, a relative humidity of 50 ± 20%, and a 12-h/12-h light/dark cycle with lights on from 07:00–19:00 h daily and were allowed free access to water and standard laboratory food (MF, Oriental Yeast Co., Ltd., Tokyo, Japan). The animals were used in the present study after habituation for 3 days.

### Plasma and brain sample collection

GM (5 mg/kg) or vehicle (70% DMSO; 0.6 mL/kg) was injected into the tail vein of rats fasted for approximately 16 h. The animals (n = 3/each point) were sacrificed at 0.25, 1, or 4 h under anesthesia with isoflurane after injection by collecting whole blood from the abdominal inferior vena cava using heparinized syringes. Blood samples were centrifuged at 1,700 × *g* and 4 °C for 15 min to obtain the plasma. The brain of each animal was immediately removed and weighed after blood collection and frozen with liquid nitrogen.

Blank plasma or brain samples (i.e., blank matrix samples) were also obtained from two normal rats after fasting for approximately 16 h by using the same procedure. These samples were used to generate the calibration curve for measuring plasma or brain GM concentrations in the LC-MS/MS or DESI-MSI analysis.

### LC-MS/MS analysis of GM and its metabolites HM-1/2 in the plasma

On the day of analysis, frozen plasma from GM-treated rats was thawed at room temperature, and 25 µL of the plasma was mixed with equal volumes of methanol and an IS vincamine (1 ng/mL) and 150 μL of ethyl acetate. To prepare the calibration curve, blank plasma and various concentrations of working solution (final plasma concentration: 0.08–2 µg/mL) were used instead of methanol and GM-treated plasma. The mixture was centrifuged at 7,000 × *g* and 4 °C for 5 min. The supernatant was dried under a stream of nitrogen gas. The dried residue was dissolved in 60 μL of the initial mobile phase of LC, and then, a 5-μL aliquot was injected into the LC-MS/MS system composed of an Agilent 1290 LC (Agilent Technologies, Santa Clara, CA, USA) and a TripleQuad6500 MS/MS (SCIEX, Framingham, MA, USA) for measuring GM and its hydroxylated forms.

The analytical conditions for LC-MS/MS were set according to previously reported methods with minor modifications^10,15^. The protonated ions ([M + H]^+^) of GM (*m/z* 367.0), HM-1/2 isomers (*m/z* 383.3), and vincamine (*m/z* 355.3) in Q1 (the first MS) or their specific fragment ions (GM: *m/z* 144.1, HM-1/2 isomers: *m/z* 160.2, vincamine: *m/z* 337.1) in Q3 (the second MS) were selected and detected by SRM using positive ion mode. Although a qualifier ion was not monitored in their analysis, the specificities of GM, HM-1/2 and vincamine in rat plasma were already confirmed by previous studies^10,14^.

The calibration curve for quantification of plasma GM concentrations was prepared using Analyst software (version 1.6.2, AB SCIEX). The plasma concentrations of HM-1/2 were expressed as relative peak area ratios of vincamine (IS) because there are currently no standard products for measuring these compounds.

### MSI analyses of GM and HM in the brain slice section

The frozen brains were used for making serial sagittal thin sliced sections (thickness 25 μm, coordinate: approximately 3.4 mm lateral from the bregma) using a Leica CM3050S cryostat (Leica Microsystems, Wetzlar, Germany). Some of the serial sections mounted on conventional glass slides were used for H&E staining to confirm the location of the brain region in the slice. In MALDI-MSI analysis, some sections were mounted on indium tin oxide-coated slides (Matsunami Glass Ind., Osaka, Japan) based on previous reports^35,36^ to obtain higher sensitivity. In DESI-MSI analysis, some sections were mounted on conventional glass slides. These tissue sections mounted on the slides were dried in a desiccator at room temperature for 10 min for MALDI-MSI analysis or 1 h for DESI-MSI analysis. In the DESI-MSI analysis, the MS/MS analysis was further performed to identify the signals of GM or its hydroxylated metabolite.

The analytical conditions of the MALDI-MSI or DESI-MSI were as follows.

#### MALDI-MSI conditions

The MALDI-MSI analysis was performed on brain slice specimens sprayed with a matrix solution (40 mg/mL 2,5-dihydroxybenzoic acid) dissolved in 50% methanol using a TM-sprayer (HTX Technologies, Chapel Hill, NC, USA). The MS analysis in the MALDI-MSI was performed using SolariX 7 T FT-ICR (Bruker Daltonics, Bremen, Germany). The targeted protonated molecule GM ([M + H]^+^: *m/z* 367.2) was detected in positive ion mode under a laser power setting of 70%, 500 laser shots, and the continuous accumulation of selected ions mode set to ON at *m/z* 360 ± 50. MALDI-MSI measurements were acquired at 200 µm spatial resolution. GM intensity detected by the MALDI-MS analysis was visualized using FlexImaging software (Bruker Daltonics).

#### DESI-MSI conditions

The MS and MS/MS analyses for DESI-MSI were performed using a Xevo G2-XS Q-Tof system (Waters, Milford, MA, USA) with an Omni Spray Ion Source 2-D (Prosolia, Indianapolis, IN, USA). In both analyses, methanol/water (98:2)solution was flowed into a capillary at a 2 μL/min flow rate. The nebulizing gas pressure was set to 0.4 MPa, the capillary voltage to 5 kV, the source temperature to 100 °C, the sampling cone to 30 V, and the scan time to 0.986 sec.

In the MS analysis, the collision energy was set to 4 eV, and mass spectra were acquired in target enhancement mode, which was set to *m/z* 367.2. In the MS/MS analysis, the collision energy was set to 20 eV, and mass spectra were acquired in target enhancement mode, which was set to *m/z* 144.2. Mass resolution and spatial resolution were >20,000 and 200 µm in the MS and MS/MS analyses.

The protonated ions of GM ([M + H]^+^: *m/z* 367.2) and HM ([M + H]^+^: *m/z* 383.2) in MS and the specific fragment ions of GM (*m/z* 144.2) and HM (*m/z* 160.2) in MS/MS were detected in positive ion mode. The intensities of GM and HM detected in the DESI-MS analysis were visualized using high-definition imaging (HDI) software (Waters, Milford, MA, USA).

### Elemental composition analysis

The theoretical MS spectra of compounds with the same chemical composition as protonated GM (C_22_H_27_N_2_O_3_) or HM (C_22_H_27_N_2_O_4_) were automatically obtained by elemental composition analysis. The intensity ratio of the target compound and its isotope and exact mass in the theoretical MS spectrum were calculated and compared with those obtained from the MS spectrum of GM or HM detected in the lateral ventricle.

### Brain distribution of the endogenous standard molecule *m/z* 366.95

For DESI-MSI analysis, an endogenous standard molecule *m/z* 366.95 was used to adjust for interscan variability and ionization response of GM signal intensity in each brain region. The homogeneity of this molecule as an endogenous internal standard was compared to an image using vincamine as an extrinsic internal standard. Briefly, a 5 µg/mL solution of vincamine dissolved in methanol/water (98:2) was added into the capillary of the DESI-MSI instrument and sprayed onto the surface of the control brain sections which were made of the brain of intact rat without taking GM. HDI software was used to visualize the intensity of the detected protonated ion ([M + H]^+^: *m/z* 355.2) of vincamine.

### Region of interest analysis in DESI-MSI

Normal intact brains were homogenized without adding a solvent using a T-10 basic Ultra-Turrax homogenizer (IKA, Staufen, Germany) to prepare a tissue-based calibration curve for quantifying GM. For brain homogenates, various concentrations of standard GM solution (final concentration: 6.25, 12.5, and 25.0 μg/g brain) or vehicle (methanol) as a control were added and rapidly frozen. Frozen sections (thickness 25 μm) of the homogenates spiked with standard GM were made using a Leica CM3050S cryostat (Leica Microsystems).

Regional intensities of GM (*m/z* 367.2) and a standard endogenous molecule (*m/z* 366.95) in brain tissue slices and homogenate slices were evaluated by the region of interest analysis using HDI software (Waters). The GM signal intensity was expressed as the average of the relative intensity ratio to the standard endogenous molecule per pixels contained in the region of interest. The regional brain concentration of GM was calculated using the tissue-based calibration curve (Fig. 3D-2).

### Statistical analysis

GM and/or HM-1/2 levels in the plasma and/or brain are presented as the mean ± standard error. A factorial analysis of these data was performed using one-way ANOVA. If the ANOVA showed a significant difference, Dunnett’s multiple comparison test was performed as a post-hoc analysis. The significance threshold was set at *P* < 0.05. All statistical analyses were performed using the SAS 9.2 software (SAS Institute, Inc., Cary, NC, USA).

### Ethics

All animal experiments were planned according to the guidelines for animal care and use of laboratory animal and approved by the Experimental Animal Ethics Committees of Tsumura & Co. (approval number and date: 17-079/February 5, 2018).

## Supplementary information

Supplementary Information.

## Data Availability

All data generated or analyzed during this study are included in this published article (and its Supplementary Information Files).
