# Supplementary material for: Brain distribution of geissoschizine methyl ether in rats using mass spectrometry imaging analysis
Source: Sci Rep. 2020 Apr 29;10:7293. doi: 10.1038/s41598-020-63474-x (PMC7190722; doi:10.1038/s41598-020-63474-x)
Supplement: Supplementary file 1 — Supplementary Information. [file 41598_2020_63474_MOESM1_ESM.docx]

**Supplementary Information**

**Brain distribution of geissoschizine methyl ether in rats using mass spectrometry imaging analysis**

Takashi Matsumoto^1*^, Yasushi Ikarashi^1^, Mikina Takiyama^1^, Junko Watanabe^1^, Mitsutoshi Setou^2,3,4^

^1)^ Tsumura Kampo Research Laboratories, Kampo Research & Development Division, Tsumura & Co., Ibaraki, Japan

^2)^ Department of Cellular and Molecular Anatomy, Hamamatsu University School of Medicine, Hamamatsu, Shizuoka, Japan

^3)^ International Mass Imaging Center, Hamamatsu University School of Medicine, Hamamatsu, Shizuoka, Japan

^4)^ Department of Systems Molecular Anatomy, Institute for Medical Photonics Research, Preeminent Medical Photonics Education & Research Center, Hamamatsu, Shizuoka, Japan

* Corresponding author:

Takashi Matsumoto, Ph.D.

Tsumura Kampo Research Laboratories,

Kampo Research & Development Division,

Tsumura & Co.

3586 Yoshiwara, Ami-machi, Inashiki-gun, Ibaraki 300-1192, Japan

Phone: +81-29-889-3852; Fax: +81-29-889-3870

E-mail: [matsumoto_takashi@mail.tsumura.co.jp](mailto:matsumoto_takashi@mail.tsumura.co.jp)


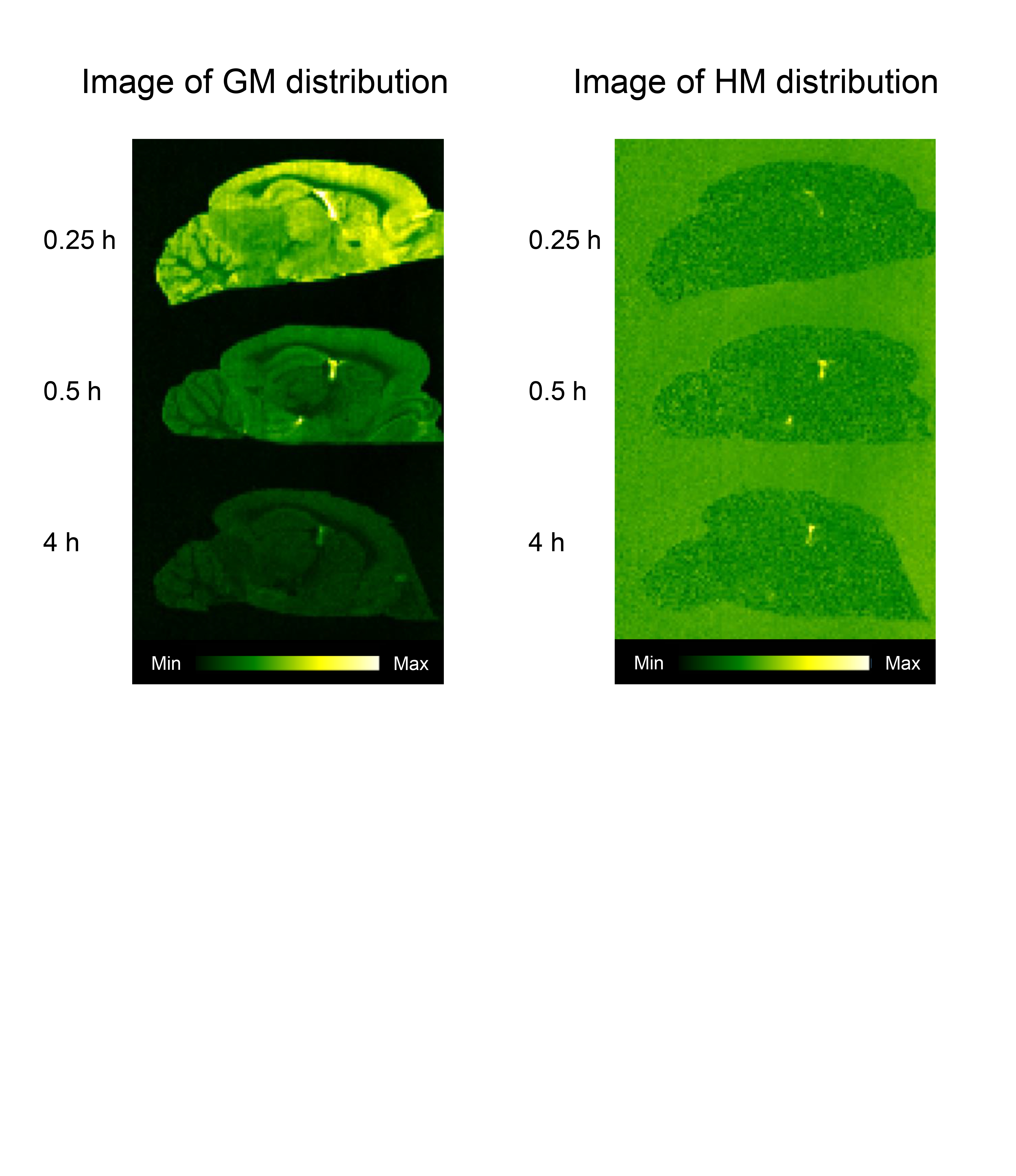


**Supplementary Figure S1. Reproducibility images for the time-dependent distributions of GM and HM.** To illustrate the reproducibility of Figures 3A and 4A by DESI-MSI analysis, this figure shows another GM and HM distribution images analyzed repeatedly in brain sections obtained from mice 0.25–4 h after injection.

**
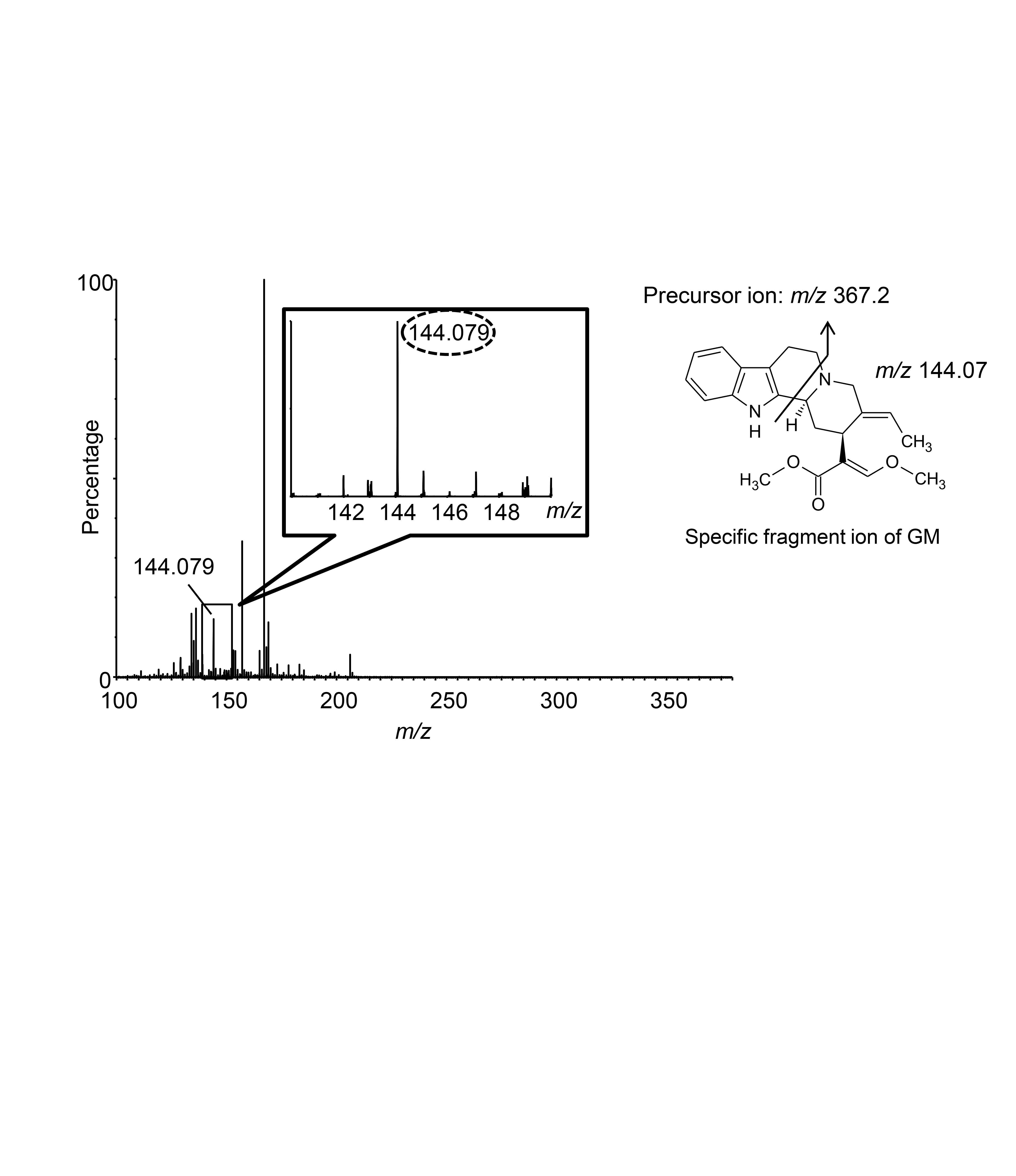
**

**Supplementary Figure S2. MS/MS spectrum of protonated GM detected in the lateral ventricle.** Specific fragment ion (*m/z* 144.079) from protonated GM ion (*m/z* 367.2) was generated by collision-induced dissociation.

**
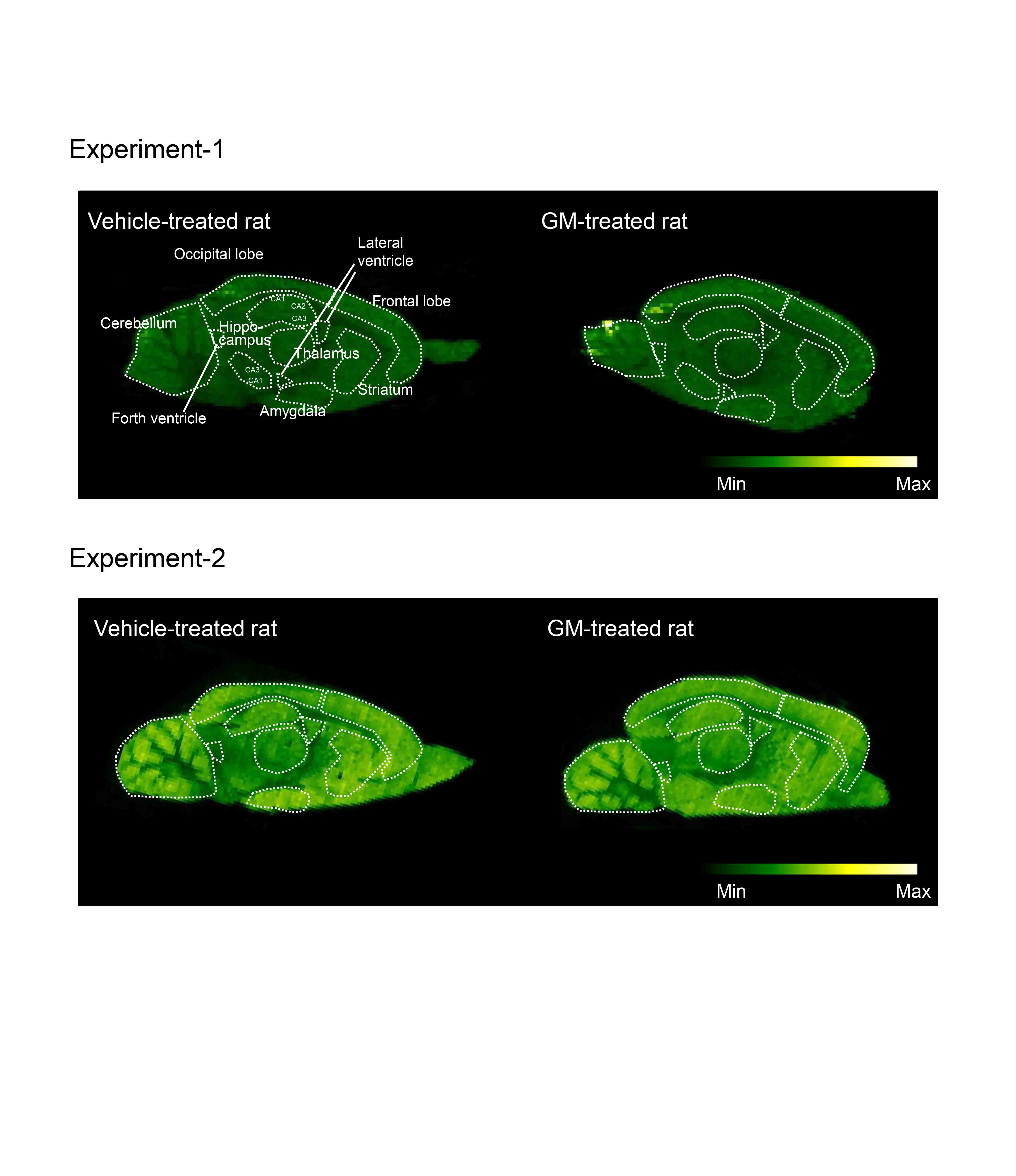
**

**Supplementary Figure S3. Detection of endogenous internal standard molecule in rat brains using DESI-MSI analysis.** Endogenous molecular ion of *m/z* 366.95 was detected stably and uniformly in the brains of vehicle- and GM-treated rats. Therefore, *m/z* 366.95 was used as an endogenous internal standard molecule to adjust the interscan variability of GM signal intensity in brain tissue.


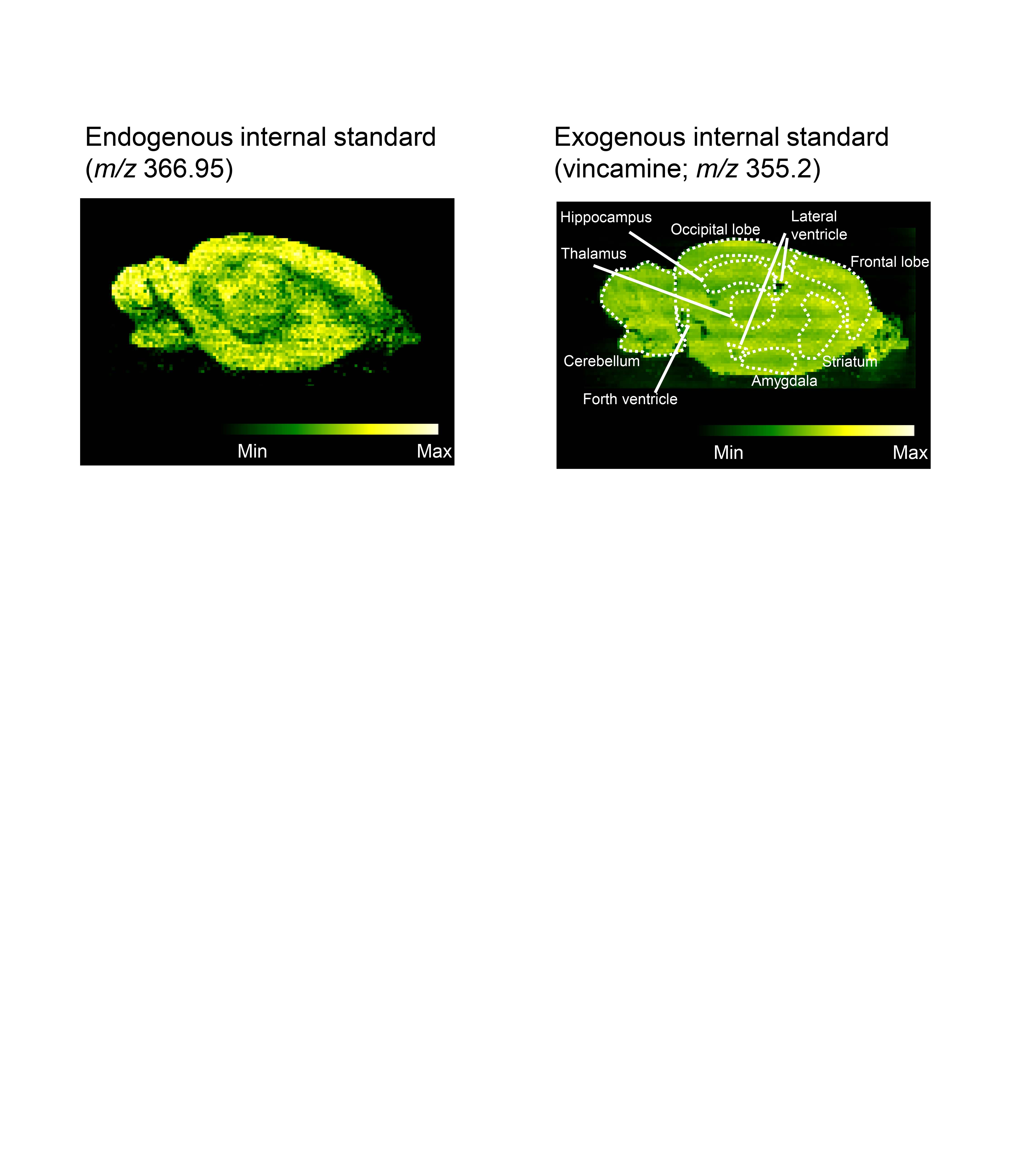


**Supplementary Figure S4. DESI-MSI images of the endogenous and exogenous molecules as internal standards in the control brain section.** In an examination of the homogeneity of the distribution of the *m/z* 366.95 molecule used as endogenous internal standard in this study, the DESI-MSI images of the endogenous *m/z* 366.95 molecule and exogenous molecule vincamine in the control brain sections were compared. The control brain sections were made of the brain of intact rat without taking GM. Vincamine solution (5 μg/mL) was added to the capillary of the DESI-MSI instrument and sprayed onto the surface of the brain section. As a result, the *m/z* 366.95 molecule was confirmed to be distributed reliably and uniformly in each region of the brain like vincamine.

**Supplementary Table S1. The intensity values of GM and the endogenous *m/z* 366.95 molecule used for drawing Figure 3E.** Figure 3E shows regional GM concentrations in 0.25 h brain images. GM concentrations were calculated using the calibration curve (y=0.8364x; y: relative intensity ratio, x: concentration of GM) shown in Figure 3D-2 and the relative intensity ratio of GM to the endogenous standard molecule per pixels contained in the region of interest. All values used in calculations of GM concentrations are summarized here.

| Region | Ion intensity of GM per pixel in ROI (×10^4^) | Ion intensity of endogenous standard molecule per pixel in ROI (×10^4^) | Ratio of GM/endogenous standard molecule | Calculated GM concentration (µg/g) |
| --- | --- | --- | --- | --- |
| Frontal lobe | 2.9 | 3.7 | 0.8 | 1.0 |
|  | 5.4 | 2.5 | 2.2 | 2.6 |
|  | 9.1 | 9.1 | 1.0 | 1.2 |
| Occipital lobe | 2.9 | 3.3 | 0.9 | 1.1 |
|  | 4.8 | 2.1 | 2.3 | 2.7 |
|  | 10.5 | 10.0 | 1.1 | 1.3 |
| Hippocampus | 2.0 | 2.9 | 0.7 | 0.8 |
|  | 4.7 | 2.5 | 1.9 | 2.3 |
|  | 10 | 11.1 | 0.9 | 1.1 |
| Striatum | 1.9 | 2.9 | 0.7 | 0.8 |
|  | 3.9 | 1.8 | 2.2 | 2.6 |
|  | 7.2 | 8.0 | 0.9 | 1.1 |
| Amygdala | 2.4 | 3.1 | 0.8 | 1.0 |
|  | 3.7 | 2.1 | 1.8 | 2.2 |
|  | 9.7 | 9.7 | 1.0 | 1.2 |
| Thalamus | 1.7 | 2.7 | 0.6 | 0.7 |
|  | 4.5 | 2.3 | 2.0 | 2.4 |
|  | 8.8 | 9.8 | 0.9 | 1.1 |
| Cerebellum | 4.2 | 6.3 | 0.7 | 0.8 |
|  | 2.4 | 1.4 | 1.7 | 2.0 |
|  | 8.7 | 9.5 | 0.9 | 1.1 |
| Lateral ventricle | 3.0 | 2.5 | 1.2 | 1.4 |
|  | 8.3 | 2.2 | 3.8 | 4.5 |
|  | 21.2 | 8.9 | 2.4 | 2.9 |
| Fourth ventricle | 4.0 | 4.0 | 1.0 | 1.2 |
|  | 3.9 | 2.1 | 1.9 | 2.3 |
|  | 26.5 | 15.4 | 1.7 | 2.0 |

ROI: region of interest.
